# A migrating fishbone

**DOI:** 10.1055/a-2233-2968

**Published:** 2024-02-07

**Authors:** Lei Shi, SaiLing Wei, Qiong Yan, Jia Xun Xie, JinYu Wu, Lisheng Wang

**Affiliations:** 1 Department of Gastroenterology, Shenzhen People’s Hospital (Second Clinical Medical College, Jinan University), Shenzhen, China; 2 556508 Gastroenterology, The Affiliated Hospital of Southwest Medical University, Luzhou, China

A 65-year-old man presented to our hospital having had epigastric discomfort for 1 month. Abdominal computed tomography (CT) demonstrated a strip of high density shadow in the medial wall of the gastric antrum ( Fig. 1 ). After a detailed medical history had been taken, the patient recalled the ingestion of fish 1 month previously.

**Fig. 1:**
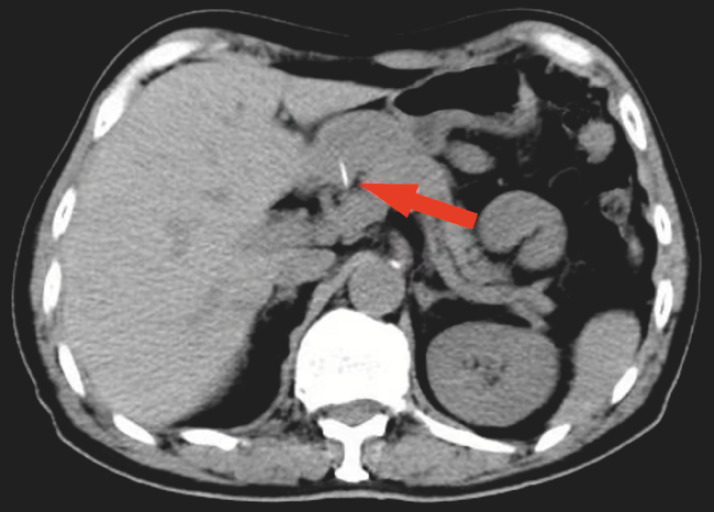
Abdominal computed tomography demonstrated a strip of high density shadow (arrow) in a patient presenting with epigastric discomfort of 1 month’s duration.

Endoscopy revealed a bulge in the gastric antrum ( Fig. 2 ) and a bulge in the duodenal bulb ( Fig. 3 ). Endoscopic ultrasound (EUS) revealed a hyperechoic strip in the muscularis propria of the stomach, with one end penetrating into the submucosal layer of the duodenal bulb ( Fig. 4 ). Therefore, a preliminary diagnosis of a foreign body that had penetrated the gastric wall was considered.

**Fig. 2:**
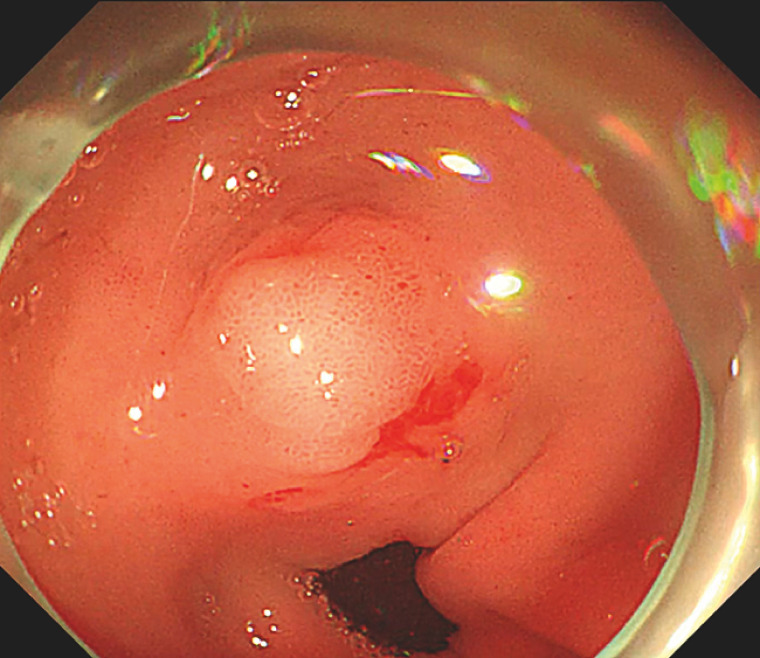
A bulge on the surface of the gastric sinus was seen at endoscopy.

**Fig. 3:**
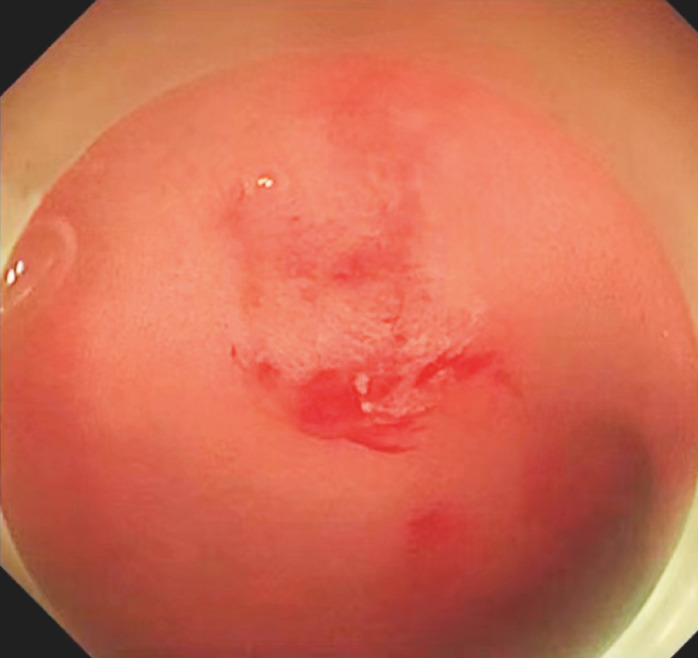
Another bulge was found on the surface of the duodenal bulb.

**Fig. 4:**
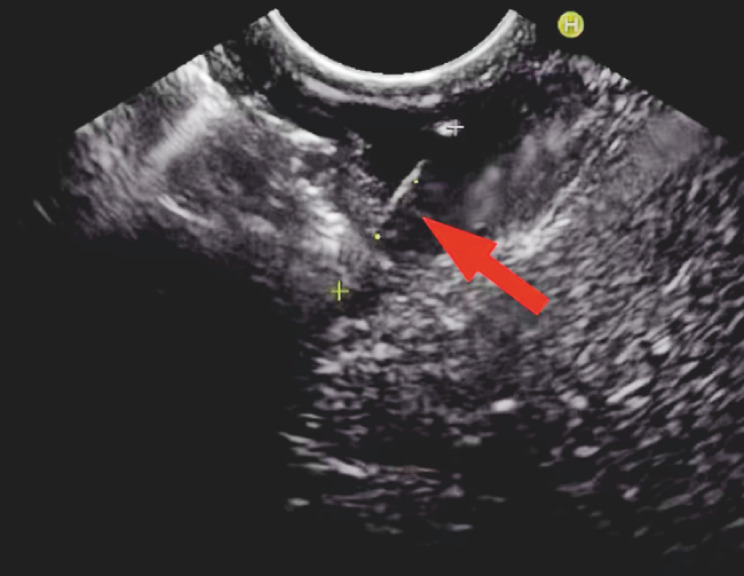
Endoscopic ultrasonography revealed the location and depth of a fishbone (arrow).

Based on these examinations, we chose to perform endoscopic submucosal dissection (ESD) through the duodenal bulb ( Video 1 ). After submucosal injection and incision of the bulbar mucosa, thermal biopsy forceps(FD-410LR; Olympus, Tokyo, Japan) were used to pull the incised edge of the mucosa. One end of the transparent fishbone was exposed in the submucosa, and was then removed using the thermal biopsy forceps ( Fig. 5 ). Finally, the mucosal incision was closed with several metal clips.

Retrieval of fishbone that had penetrated the wall of the gastric sinus and migrated to the duodenal bulb, by endoscopic submucosal dissection.Video 1

**Fig. 5:**
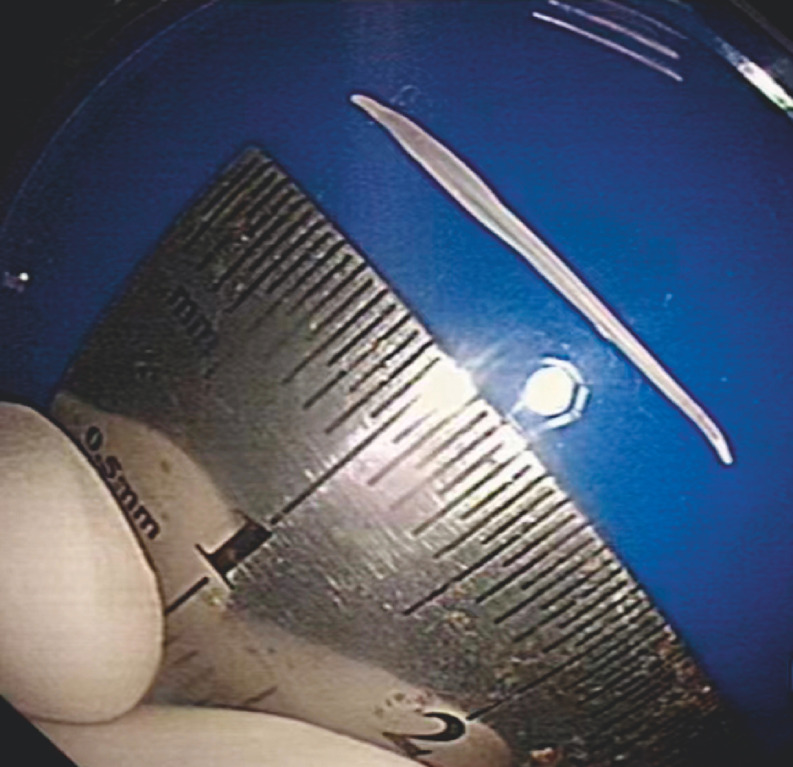
The 1.8-cm fishbone, extracted using the thermal biopsy forceps.

Penetration by foreign bodies in the stomach generally occurs in the gastric antrum, and the foreign body can then penetrate further into the submucosa, or the muscular layer, or the abdominal cavity 1 . It is rare for a foreign body to penetrate through the gastric antrum to the duodenal bulb. In the present case, the fishbone that pierced the gastric sinus may have been pushed to the duodenal bulb by the peristalsis of the gastric sinus. Therefore, when considering penetration of foreign bodies in the gastric antrum, it may also be important to observe the duodenal bulb. In similar cases, detailed inquiry into medical history and CT combined with EUS examination can help to clarify the diagnosis and determine treatment plans 2 . ESD may be the preferred option for removal, allowing the patient to avoid laparoscopic or open surgery 3 .

Endoscopy_UCTN_Code_CCL_1AB_2AF

## Notes

Endoscopy E-Videos https://eref.thieme.de/e-videos
*E-Videos* is an open access online section of the journal *Endoscopy* , reporting on interesting cases and new techniques in gastroenterological endoscopy. All papers include a high-quality video and are published with a Creative Commons CC-BY license. Endoscopy E-Videos qualify for HINARI discounts and waivers and eligibility is automatically checked during the submission process. We grant 100% waivers to articles whose corresponding authors are based in Group A countries and 50% waivers to those who are based in Group B countries as classified by Research4Life (see: https://www.research4life.org/access/eligibility/ ). This section has its own submission website at https://mc.manuscriptcentral.com/e-videos .
